# Nesprins and Lamins in Health and Diseases of Cardiac and Skeletal Muscles

**DOI:** 10.3389/fphys.2018.01277

**Published:** 2018-09-07

**Authors:** Alexandre Janin, Vincent Gache

**Affiliations:** ^1^CNRS UMR5310, INSERM U1217, Institut NeuroMyoGène, Université Claude Bernard Lyon 1, Université de Lyon, Lyon, France; ^2^Laboratoire de Cardiogénétique Moléculaire, Centre de Biologie et Pathologie Est, Hospices Civils de Lyon, Bron, France

**Keywords:** LINC complex, nuclear envelope, lamins A/C, *LMNA*, nesprins, human diseases

## Abstract

Since the discovery of the inner nuclear transmembrane protein emerin in the early 1990s, nuclear envelope (NE) components and related involvement in nuclei integrity and functionality have been highly investigated. The NE is composed of two distinct lipid bilayers described as the inner (INM) and outer (ONM) nuclear membrane. NE proteins can be specifically “integrated” in the INM (such as emerin and SUN proteins) or in the ONM such as nesprins. Additionally, flanked to the INM, the nuclear lamina, a proteinaceous meshwork mainly composed of lamins A and C completes NE composition. This network of proteins physically interplays to guarantee NE integrity and most importantly, shape the bridge between cytoplasmic cytoskeletons networks (such as microtubules and actin) and the genome, through the anchorage to the heterochromatin. The essential network driving the connection of nucleoskeleton with cytoskeleton takes place in the perinuclear space (the space between ONM and INM) with the contribution of the LINC complex (for Linker of Nucleoskeleton to Cytoskeleton), hosting KASH and SUN proteins interactions. This close interplay between compartments has been related to diverse functions from nuclear integrity, activity and positioning through mechanotransduction pathways. At the same time, mutations in NE components genes coding for proteins such as lamins or nesprins, had been associated with a wide range of congenital diseases including cardiac and muscular diseases. Although most of these NE associated proteins are ubiquitously expressed, a large number of tissue-specific disorders have been associated with diverse pathogenic mutations. Thus, diagnosis and molecular explanation of this group of diseases, commonly called “nuclear envelopathies,” is currently challenging. This review aims, first, to give a better understanding of diverse functions of the LINC complex components, from the point of view of lamins and nesprins. Second, to summarize human congenital diseases with a special focus on muscle and heart abnormalities, caused by mutations in genes coding for these two types of NE associated proteins.

## Introduction

The Nuclear Envelope (NE) is composed of two distinct lipid bilayers so called inner (INM) and outer (ONM) nuclear membrane. This particular membrane architecture allows protection of chromatin from the rest of the cell by a physical separation between cytoplasm and nucleoplasm (Osorio and Gomes, 2013). Transmembrane proteins embedded in these two joined-membranes provide an architectural support to the nucleus as well as a physical bridge between nucleoskeleton and cytoskeleton. Physical coupling of these two skeletons is based on the so-called LINC complex (for Linker of the Nucleoskeleton to the Cytoskeleton). It couples, in the perinuclear space, proteins localized at the ONM and containing KASH domains (Klarsicht/Anc-1/Syne homology) with proteins localized at the INM and containing SUN domains (Sad1/UNC-84 homology) (Tapley and Starr, 2013). To date, six KASH domain-containing proteins have been described: nesprins-1, -2, -3, -4, KASH5 and lymphoid-restricted membrane protein (LRMP), respectively, encoded by *SYNE1, SYNE2, SYNE3, SYNE4, KASH5*, and *LRPM* genes (Behrens et al., 1994; Zhang et al., 2001; Wilhelmsen et al., 2005; Roux et al., 2009). SUN proteins are embedded in the INM and interact using their N-terminus domain with nuclear pore complex (NPC) and lamins (Padmakumar et al., 2005; Haque et al., 2006). Conversely, C-terminus domain is located in the perinuclear space and interacts with nesprins, which are embedded in the ONM (Padmakumar et al., 2005; Crisp et al., 2006; Haque et al., 2006; Ketema et al., 2007; Horn et al., 2013). This LINC complex play a role in diverse specialized cellular activities such as nuclear morphology maintenance, nuclear positioning, genes expression and cell signaling (Crisp et al., 2006; Lombardi and Lammerding, 2011; Mellad et al., 2011; Stroud et al., 2014). At the nucleoplasmic side, the LINC complex interacts with the nuclear lamina, a network of intermediate filaments just beneath INM and mainly composed of two different groups of lamin: A-type (lamins A and C) and B-type lamins (lamins B1 and B2) (Fisher et al., 1986; McKeon et al., 1986; Peter et al., 1989).

In this review, we will summarize functional diversities of proteins associated to the LINC complex with a particular focus on lamin A/C and nesprins. We will recapitulate known muscular and cardiac abnormalities induced by mutations in those genes and will discuss our recent advances in related pathogenesis.

## Nesprins and Lamins as Components of the LINC Complex

**FIGURE 1:**
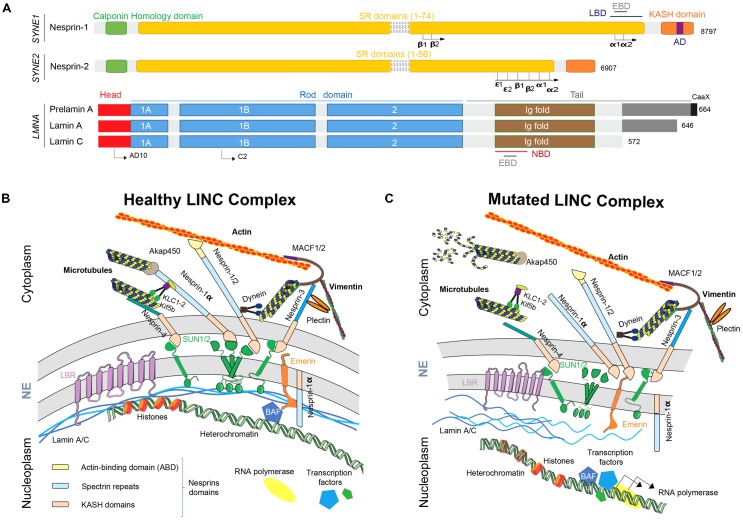
Nesprins and lamins as part of the LINC complex. **(A)** Nesprins and lamins isoforms structures. AD, adaptive domain; EBD, emerin binding domain; KASH domain, Klarsicht/ANC-1/Syne Homology; LBD, lamin binding domain; NBD, nesprin binding domain; SR, spectrin repeats. **(B)** The healthy LINC complex and its interactors. Nesprins play multiple functions at ONM but also at INM. Full-length nesprins take part at the ONM to the bridge linking the nucleus to the actin cytoskeleton and shorter isoforms, such as nesprin-1a, interact via Akap450 and KLC-1/2 with microtubules. At the same time, smaller isoforms interact with the nucleoskeleton at INM. **(C)** When a mutation affects *LMNA* or *SYNE* genes, the disruption of the LINC complex participates to the physiopathogenesis of the diseases by structural defects, nuclear migration and anchoring defects and cell signaling pathways and gene expression regulation defects. NE, nuclear envelope; LINC, linker of nucleoskeleton to cytoskeleton; ONM, outer nuclear membrane; INM, inner nuclear membrane; KASH domain, Klarsicht/ANC-1/Syne homology; SUN, Sad1p/UNC84; BAF, barrier to auto-integration factor.

In human, full-length nesprin-1 and nesprin-2, which are the ubiquitously expressed giant nesprins isoforms, are respectively, with a molecular weight of 1 MDa (146 exons) and 800 kDa (116 exons), the second and the third largest described proteins, after the “untouchable” titin (4.2 MDa) (Zhang et al., 2001). Nesprins-1 and -2 are ubiquitous proteins, commonly described as ONM components and composed of three major domains: (i) a Calponin Homology (CH) domain, also called Actin-Binding Domain (ABD) located in the N-terminus side that binds to the actin cytoskeleton; (ii) a long central rod domain composed of multiple spectrin repeats (SR) (respectively, 74 and 56 SR in nesprin-1 and -2) and that supports interactions with other proteins such as emerin with the Emerin Binding Domain (EBD) or lamins with the Lamin Binding Domain (LBD) and finally (iii) a C-terminus KASH domain embedded in the ONM (Zhang et al., 2001; Rajgor and Shanahan, 2013; **Figure 1A**). An additional domain has been described and called “adaptive domain” (AD). This highly conserved domain is located at the C-terminus extremity and is crucial for structural stabilization of SR (Simpson and Roberts, 2008; Zhong et al., 2010).

Numerous isoforms of nesprin-1 and -2 are produced by alternative initiation/termination of transcription and/or alternative splicing of *SYNE1* and *SYNE2* genes. These isoforms have a tissue-specific expression and exhibit different subcellular locations, such as actin stress-fibers, focal adhesions or microtubules (Rajgor et al., 2012; Duong et al., 2014). As previously described, full-length isoforms are located at the ONM and, thanks to their KASH domains, interacts in the perinuclear space with SUN1/2. This layout allow the interaction with both actin and microtubule networks through associated molecular motors such as dynein, kinesins light chain (KLC-1/2) and/or microtubule-mediated nucleation factor, such as akap450 (Wilson and Holzbaur, 2015; Gimpel et al., 2017). Interestingly, some isoforms such as full-length nesprin-2 and nesprin-1α, a small isoform, could also localize at the INM leading to the direct interaction with emerin, SUN1/2 and/or lamin A/C (**Figure 1A**; Zhang, 2005; Holt et al., 2016).

Nesprin-1 and -2, especially small isoforms, nesprin-1α_2_ and -2α_1_, are particularly highly expressed in skeletal and cardiac muscles (Duong et al., 2014). Despite the fact that *SYNE1* and *SYNE2* genes are about 60% homologous, nesprin-1 is more conserved than nesprin-2 (Zhang et al., 2001). However, ABD domains and C-terminus part of SR domains are highly conserved, especially in nesprin-1α_2_, -2α_1_ and -2𝜖_2_, short skeletal muscle-specific and cardiac-specific isoforms. These conserved domains suggest that these isoforms play a similar role in skeletal muscle and cardiac cells physiology (Autore et al., 2013). Nesprin-3, also ubiquitously expressed, was first discovered associated with the ABD of the cytoskeletal linker protein plectin (Wilhelmsen et al., 2005; Ketema et al., 2013). Plectin acts as a mechanical linker between intermediate filaments networks and various cellular structures and is crucial for myofibres integrity. Plectin deficiency has been shown to affect chromatin structure and some genes expression mediating mechanotransduction. Nesprin-3 also interact with ABD of MACF1/2 (Microtubule-Actin Cross Factor 1/2) thanks to its unique spectrin repeat domain and contribute to vimentin accumulation at the vicinity of the nucleus (Wilhelmsen et al., 2005; Ketema et al., 2013; **Figure 1B**). Nesprin-4 is surprisingly exclusively produced by secretory epithelia and mechanosensory cochlea hair cells (Roux et al., 2009; Horn et al., 2013). *KASH5* is only expressed in meiotic cells (Stewart and Burke, 2014). And, finally, *LRPM* is expressed in some taste cells of mammalian tongues and plays a crucial role in signal transduction by binding IP3 receptors (Shindo et al., 2010). To resume, nesprins-1 and -2 and related isoforms form a strong physical links allowing connection between nucleoskeleton and cytoskeleton including microtubules network, actin and intermediate filaments.

The nuclear lamina is composed of class-V intermediate filaments proteins called “nuclear lamins.” Several isoforms have been described in mammals, divided in two groups: A and B-type lamins (Dechat et al., 2008). A-type lamins are encoded by *LMNA* gene, located on chromosome 1, producing two major isoforms in somatic cells: lamin-A and lamin-C (**Figure 1A**). These isoforms are expressed in the majority of differentiated cells, although absent in early stages of embryonic development (Dechat et al., 2010). These two isoforms are produced by an alternative splicing in exon 10. Thus, N-terminus part of lamin-A and -C are similar until the 566th amino-acid and then, lamin-C shows five specific amino-acids in the C-terminus part while lamin A exhibit a specific domain encoded by exons 11 and 12 of *LMNA* gene (**Figure 1A**; Furukawa et al., 1994). As the other intermediate filaments, nuclear lamins form dimers by their central rod domain (Stuurman et al., 1998; Herrmann and Aebi, 2016). Additionally, three isoforms of B-type lamins have been described: lamin B1 encoded by *LMNB1* gene and lamins B2 and B3, encoded by *LMNB2* gene (Dittmer and Misteli, 2011). Nuclear lamin A and C are able to bind both nesprin-1α (Mislow et al., 2002) and nesprin-2 (Yang et al., 2013), heterochromatin (Bruston et al., 2010) and emerin (Sakaki et al., 2001), that itself bind transcription factors and DNA-binding proteins such as barrier to autointegration factor (BAF) (Burke, 1990; Yuan et al., 1991; Furukawa et al., 2003; Segura-Totten and Wilson, 2004; Dialynas et al., 2010; Jamin and Wiebe, 2015; **Figure 1B**).

This association between nesprins at the ONM through SUN proteins at the INM and lamins at the vicinity of INM is the key place in cells where mechanotransduction occurs. This process allows cytoplasmic mechanical forces, initiated through the dynamics and forces generated by different cytoskeletons (actin, microtubule, and intermediate filaments) to cross the nuclear membrane and influence the transcriptional state of the cell. This transduction can alter transcriptional efficiency through chromatin organization and regulation of epigenetics markers as well as processes linked to DNA replication (Tajik et al., 2016; Kirby and Lammerding, 2018; Wang et al., 2018). The respective role of alternative pathways in mechanical transduction during lifetime of cells remains to be determined. Interestingly, in post-mitotic conditions such as muscle cells, proteins of the LINC complex, especially nesprins and lamins emerged as a fundamental node in the crossroad of muscle pathogenesis.

## Roles of Nesprins and Lamins in Muscle Physiology

Cardiac and skeletal muscles are both striated muscles composed of repeated units called sarcomeres. Sarcomeres of both types of muscle are an arrangement of alternating thick and thin filaments resulting in a striated cell pattern. Cardiomyocytes are mononucleated cells with a centrally localized nucleus and form, together with associated cardiomyocytes, a syncytium-like via intercalated disks that link membranes of contiguous cardiomyocytes, providing synchronized contraction. Skeletal muscle is an association of large multinucleated cells with peripheral nuclei called myofibers, resulting from the fusion of specialized cells, myocytes (Tedesco et al., 2010; Chal and Pourquié, 2017). Prior to fusion, myoblasts are highly proliferative, but exit the cell cycle at the onset of myoblasts differentiation and become myocytes, specialized cells possessing the potential to fuse with each other (Yin et al., 2013). As myocytes fuse, they form multinucleated syncytium: myotubes. As myotubes mature into myofibers, contractile units (sarcomeres) are formed. A fully developed muscle is made of an association of myofibers (Sanger et al., 2010; Lindskog et al., 2015). Interestingly, myonuclei positioning is dramatically different between skeletal and cardiac muscles (peripheral vs. central). One could propose that LINC complex composition or arrangement and binding partners will be different between these two tissues. However, evidences about these differences are still lacking and will probably highlight alternative mechanisms in these two systems.

The LINC complex integrity has been shown to be crucial in myonuclei positioning and movements during both fusion of myoblasts and differentiation processes into myotubes/myofibres (Dupin et al., 2011; Gundersen and Worman, 2013; Wilson and Holzbaur, 2015; Stroud et al., 2017). More precisely, nesprin-1 is known to be critical in myonuclear anchoring in skeletal muscle (Zhang et al., 2007b; Puckelwartz et al., 2009) allowed by the bridge between myonuclei and actin cytoskeleton (Padmakumar et al., 2004; Zhang et al., 2007b, 2010; Puckelwartz et al., 2009; Banerjee et al., 2014). Proper nuclear positioning and movements in muscle is mainly mediated by cytoskeletal networks (microtubules and actin) and is known to depend on members of the LINC complex in association with nucleoskeleton components (Metzger et al., 2012; Chang et al., 2015; Wilson and Holzbaur, 2015; Gache et al., 2017; Roman et al., 2017). Both full-length nesprin-1 and nesprin-2 interact with Kif5b molecular motors through a conserved motif located in their C-terminus part of kinesins light chain KLC-1/2. This interaction is crucial for nuclear movement and positioning in differentiated myotubes (Metzger et al., 2012; Wilson and Holzbaur, 2015; Zhou et al., 2017). Moreover, Nesprin-1α is now known to be able to recruit Akap450 to the NE. This recruitment is crucial for nuclear positioning and microtubule organization during myogenesis by switching the Microtubule Organizing Center (MTOC) to the NE (Gimpel et al., 2017). Additionally, differential expression kinetic of alternative nesprins isoforms occurs during muscle differentiation (Randles et al., 2010): in proliferative myoblasts, full-length nesprin-1 isoform is expressed but not nesprin-1α_2_ while during myogenesis, full-length nesprin-1 and nesprin-1α_2_ are both up-regulated (Holt et al., 2016; Stroud et al., 2017; Zhou et al., 2017). In human vascular smooth muscle cells (VSMCs), in addition to the NE localization, nesprin-2 is also present in lamellipodia, focal adhesions and filopodia (Zhang, 2005). Moreover, in human skeletal muscle cells, nesprin-2 isoforms had been shown to relocalized at sarcomeres, with a close association with titin (Zhang, 2005). Additionally, *in vivo*, nesprin-2 can supplant nesprin-1 at the NE (Randles et al., 2010). Finally, nesprins involvements in cardiomyocytes functionality and during cardiac cell differentiation are for now limited mainly because of technical issues (*i.e.*, difficulties to isolate and purify mature cardiomyocytes, absence of cardiac stem cells and regeneration process…). Nonetheless, it has been shown that nesprin-1 knockdown alter differentiation of embryonic stem cells in mature cardiomyocytes in mouse model (Gao et al., 2012) and that nesprins-1 and -2 ablation cause cardiomyopathy in mice with an altered nuclear morphology and altered mechanotransduction (Banerjee et al., 2014).

Plectin, a cytoskeletal protein interacting with nesprin-3, acts as a mechanical linker between intermediate filaments networks and various cellular structures and is crucial for myofibres integrity. Plectin deficiency has been shown to affect chromatin structure, specific genes expression mediating mechanotransduction but also mitochondrial network organization with the upregulation of Mfn-2, the mitochondrial fusion-associated protein mitofusion-2 (Staszewska et al., 2015; Winter et al., 2015).

In the same manner, lamins exert their function thanks to their interaction with numerous lamin-binding partners. Over 100 putative binding proteins are predicted with a high variety of functions: mechanotransduction, cell migration and differentiation (Gerace and Tapia, 2018). Crucial role of lamins reside in the structural support gave to the nuclei in cell, especially in contractile tissues. This role is mediated by a structure and assembly of lamins based on an α-helicoidal coiled coil structure assembled into fibers (Gruenbaum and Foisner, 2015). This typical network pattern provides resistance to contractile cells, such as in cardiomyocytes and muscle fibers, which are subjected to physical forces (Carmosino et al., 2014). For example, lamin A/C, by monitoring nuclei membrane rigidity, is determinant in the control of myonuclear movement to the periphery of myofibers (Roman et al., 2017). As discussed after, this role is the foundation of “mechanical stress hypothesis,” also called “structural hypothesis” about the physiopathological mechanisms underlying *LMNA* mutations. The second crucial role of lamins is their interactions with chromatin, signaling molecules and transcription factors (Herrmann et al., 2007). This interplay with lamin A/C, but also with other LINC complex components or interactors (signaling molecules or transcription factors) is an important mechanism to explain gene expression regulation by NE proteins (Robson et al., 2016; Kirby and Lammerding, 2018; Wang et al., 2018). Numerous studies have highlighted this regulatory role of lamins in signaling pathways such as MAPK (Muchir et al., 2007), Akt/mTOR (Choi et al., 2012; Ramos et al., 2012), TGFα (Arimura et al., 2005; Cohen et al., 2013), BMP (Janin et al., 2018), YAP (Bertrand et al., 2014), and major apoptosis signaling pathways (Lu et al., 2010). Lamins A/C have also a crucial role for proper skeletal muscle stem cells differentiation: primary *Lmna*-null myoblasts exhibit altered expression of differentiation regulators such as MyoD1 or desmin (Frock et al., 2006). Moreover, lamin A/C inactivation has been shown to severely impair Serum Response Factor (SRF) pathway, which control genes coding for actin cytoskeleton (Vartiainen et al., 2007; Ho et al., 2013) and for sarcomeric proteins (Balza and Misra, 2006). This characteristic will also contribute to control physiopathological and tissue-specific mechanisms underlying in *LMNA* mutations. Finally, lamins have been shown also to play a role in autophagy and probably in senescence. In *Lmna^-^*^/^*^-^* mice, hyperactivation of mTORC pathway is responsible for autophagy inhibition and, thus, disease progression. This inhibition could lead to energy deficit and accumulation of damaged and toxic proteins and/or organelles (Ramos et al., 2012).

## Nesprins and Lamins Mutations in Human Diseases

### Mutations in *SYNE* Genes

Mutations in *SYNE1*, coding for nesprin-1, have been reported to cause neurodegenerative diseases such as “Autosomal Recessive Cerebellar Ataxia type 1” (ARCA1), also called “Autosomal recessive ataxia, Beauce type” (Gros-Louis et al., 2007). In patients, homogenous phenotype was firstly reported resulting in a relatively pure and slowly progressive cerebellar ataxia with dysarthria and rare extra-neurological features and an onset at adult age. This disease was mostly identified in French and Canadian families (Dupré et al., 2007; Gros-Louis et al., 2007; Noreau et al., 2013). Later, thanks to molecular diagnosis technics improvement, diffuse pure cerebellar ataxia was challenged. Exploration of large *SYNE1* mutations cohort in European and non-European patients show that resulting phenotype is wider and encompassed neurological and non-neurological symptoms. To note, in this study, 81% of the patients exhibited additional non-cerebellar features and a multisystemic phenotype (Synofzik et al., 2016). Clinical spectrum encompasses upper and lower motor neuron disease, brainstem abnormalities and musculoskeletal features (kyphosis, scoliosis, *pes cavus*, or contractures) (Izumi et al., 2013; Synofzik et al., 2016; Algahtani et al., 2017). Similarly, onset age is variable from an early onset for some complex neurodegenerative syndromes to adult-age onset for pure cerebellar ataxia syndromes. In most cases, non-sense or intronic mutations causing premature termination could be highlighted (Synofzik et al., 2016). Thus, different nesprin-1 isoforms could be affected by these mutations, partially explaining the variability in observed phenotypes (Cartwright and Karakesisoglou, 2014; Razafsky and Hodzic, 2015). This observation is consistent with evidences in *Syne1* mutant mice model. In *Syne1^-^*^/^*^-^* mice, in which the C-terminal SR domains of nesprin-1 were ablated (Zhang et al., 2010), young mice exhibit growth retardation, increased body weight variability and exercise intolerance. In contrast, in *Syne1*^ΔKASH^, in which the KASH domain was deleted, mice exhibit normal viability but formation of both synaptic myonuclei clusters and non-synaptic myonuclei organization in skeletal muscle was impaired (Zhang et al., 2007b).

Additionally, *SYNE1* polymorphism had been associated, using genome-wide association studies (GWAS) in depression, bipolar disorders and autism and recognized as a risk factor in schizophrenia (Green et al., 2013). Thanks to whole-exome sequencing experiments, a recurrent mutation in *SYNE1* (p.Leu3206Met) had been identified as a autism-spectrum disorder candidate gene with a recurrent mutation (Yu et al., 2013).

In musculoskeletal and cardiac diseases, *SYNE1* and *SYNE2* mutations had been associated with Autosomal-Dominant Emery-Dreifuss Muscular Dystrophy (AD-EDMD) and EDMD-like phenotypes (Zhang et al., 2007a; Koch and Holaska, 2014; Fanin et al., 2015). These myopathies are usually clinically associated with three main symptoms: (i) early contractures of elbows flexors, achilles’ tendons and spine rigidity, (ii) slowly progressive muscle wasting and weakness (initiated at the humeroperoneal stage and diffuse impairment later), and (iii) cardiac misfunctionality characterized by an association of conduction defects and dilated cardiomyopathy (Koch and Holaska, 2014). Cardiac defects occur in late stages of diseases but become the main causes of EDMD patients mortality (Helbling-Leclerc et al., 2002). Mutations in *SYNE1* are also associated with pure cardiac phenotype: dilated cardiomyopathy (DCM) with conduction system defects (Puckelwartz et al., 2010; Zhou et al., 2017). Mutations in *LMNA* and *EMD* occur in nearly 40% of EDMDs (Bonne et al., 2003). Interestingly, mutations in associated partners of lamins and emerin such as nesprins-1/2 or SUN proteins have been also described in EDMD and EDMD-like phenotypes. In 2007, 190 patients suffering from EDMD or EDMD-like phenotypes without mutations in *LMNA* and *EMD* genes were analyzed regarding sequence of *SYNE1* and *SYNE2* genes. Six variants were identified, absent from control population and showing a segregation pattern compatible with an autosomal dominant inheritance (Zhang et al., 2007a). More than half of identified and characterized *SYNE1* and *SYNE2* mutants are located in the C-terminus part of full-length nesprins-1 and -2 (Zhang et al., 2007a; Attali et al., 2009; Puckelwartz et al., 2010; Taranum et al., 2012; Fanin et al., 2015; Baumann et al., 2016; Holt et al., 2016; Chen et al., 2017; Haskell et al., 2017; Wu et al., 2017; Zhou et al., 2017). In those contexts, abnormalities in nuclear morphology are frequently observed in mutated cells (commonly fibroblasts or lymphoblasts) such as altered nuclear shape, micronuclei or fragmented nuclei (Zhang et al., 2007a; Haskell et al., 2017). These observations are frequently associated with mislocalization of LINC complex components or interactors such as lamins A/C, SUN 1/2 proteins or kinesin light chain KLC-1/2, bridging cargoes to heavy chain kinesin such as Kif5b and thus to microtubules network (Zhang et al., 2007a; Chen et al., 2017; Zhou et al., 2017). Moreover, impaired fusion between myoblasts in the time course of differentiation process had been described using C2C12 cells infected with DCM-associated nesprin-1 mutations (Zhou et al., 2017). Finally, centralized myonuclei and increased variability in cross-sections area of myofibers (CSA) had been described in muscle biopsies from EDMD patients (Fanin et al., 2015; Chen et al., 2017). Partial genotype-phenotype relationship could be highlighted with *SYNE1* and *SYNE2* mutations. Most mutations causing muscle phenotype are heterozygous missense located in the C-terminus part of full-length nesprin that also belongs to nesprin-1α_2_, a muscle specific isoform (Zhou et al., 2017). Mutations affecting CNS development are located all along *SYNE1* gene (Synofzik et al., 2016). Most of them are non-sense mutations and induced premature termination codon resulting in truncated proteins or absence of nesprin-1/2 due to nonsense-mediated decay of the mutant mRNA (Zhou et al., 2018). *SYNE1* mutations are also involved in Arthrogryposis Multiplex Congenital (AMC) or simply arthrogryposis, a group of diseases with an overall prevalence of one in 3,000 live births, characterized by congenital joint contractures and reduced fetal movements (Baumann et al., 2016). Finally, some emerging role of nesprin-1 in the formation of striated F-actin-based filaments (Packard et al., 2015) or in *Drosophila*, in the control of glutamate receptors density, specifically at the neuromuscular junction (Morel et al., 2014) suggests that *SYNE1* mutations could be highlighted in other neuromuscular junction diseases.

### Mutations in *LMNA* Gene

Laminopathies is the dedicated term to call a group of rare genetic disorders caused by mutations in genes encoding proteins of the nuclear lamina: *LMNA*, coding for lamins A and C, and *LMNB1* and *LMNB2*, coding for lamins B. Laminopathies caused by *LMNA* mutations are initially defined on clinical criteria’s and is mostly characterized by an autosomal dominant inheritance (Bertrand et al., 2011). Laminopathies include a group of diseases with a predominant skeletal muscle and/or heart involvement, such as the autosomal dominant form of EDMD (EDMD-2 or AD-EDMD), the autosomal recessive form of EDMD (EDMD-3), a dilated cardiomyopathy phenotype associated with conduction defects (DCM-CD), Congenital Muscular Dystrophy (L-CMD or *LMNA*-CMD) and Limb-Girdle Muscular Dystrophy 1B (LGMD1B) (Bönnemann et al., 2014). Laminopathies also include diseases with extra-muscular phenotypes such as Dunningan-type Familial Partial Lipodystrophy (FPLD), atypical Werner syndrome, Charcot-Marie-Tooth syndrome 2B1 (CMT2B1), and Hutchinson-Gilford Progeria Syndrome (HGPS) (Shackleton et al., 2000; De Sandre-Giovannoli et al., 2002, 2003; Bonne and Levy, 2003; Bönnemann et al., 2014). Although other tissues are affected in theses diseases, muscle and heart abnormalities are the most frequently observed (Janin et al., 2017).

Skeletal muscle laminopathies share common symptoms: joint contractures affecting predominately elbows (rigidity), ankles and neck and developing toward muscle weakness, life-threatening cardiac conduction defects and dilated cardiomyopathy (Lattanzi et al., 2011). EDMD2, DCM-CD, and L-CMD are overlapping each other and are now considered as a clinical continuum of an unique disease as three phenotypes could be caused by the same mutation and could exist in the same family by variable expressivity (Lattanzi et al., 2011; Janin et al., 2017; **Table 1**). In laminopathies, serum creatine kinase activity is usually normal or slightly increased (Mercuri et al., 2004). Electromyography and histological analysis usually show unspecific patterns, thus, muscle biopsy is not necessary for diagnosis of phenotype with typical clinical abnormalities (Menezes et al., 2012). Magnetic Resonance Imaging (MRI) of muscle could help differential diagnosis with other myopathies exhibiting fatty infiltration of medial gastrocnemius and vastus muscles in *LMNA*-related myopathies (also called *LMNA*-RD) (Carboni et al., 2010, 2013; Mercuri et al., 2010; Menezes et al., 2012).

**Table 1:** Clinical and genetics comparison of *LMNA*-RD myopathies.

	EDMD-2	LGMD-1B	L-CMD
Age of onset	2nd to 3rd decade	3rd to 4th decade	Congenital onset
Muscular weakness	Scapulo-humero-peroneal distribution	Pelvic and scapular girdles	Axial (dropped head syndrome) or severe and diffuse
Contractures	+++ (elbow)	+	(spine, hips, knees, Achille tendons)
Heart abnormalities	Invariable with age, after skeletal muscle phenotype Conduction defects +/- dilated cardiomyopathy	Invariable with age	Cardiac conduction defects
Respiratory phenotype	Rare	Rare	Very frequent
Loss of independent ambulation	Rare	Rare	Very frequent
Axial involvement	Frequent	Rare	Frequent
Facial damages	Rare	Very rare	Very rare
Scoliosis	Frequent	Rare	Frequent
Type of most frequently observed *LMNA* mutations	Missense	Frameshift	Missense

*EDMD, emery-dreifuss muscular dystrophy; CMD, congenital muscular dystrophy; LGMD1B, limb-girdle muscular dystrophy 1B.*

The first described muscular phenotype related to *LMNA* mutations was EDMD-2, so called after the identification of EDMD-1 or X-linked EDMD, a phenotype caused by mutation in *EMD* coding for emerin, a inner nuclear membrane protein (Bione et al., 1994). EDMD-2 is also clinically characterized by a triad of symptoms: (i) early elbows, ankles and neck contractures with muscle weakness and wasting affecting first the scapulo-humero-peroneal area, (ii) dilated cardiomyopathy, and (iii) heart conduction system defects with high risk of sudden cardiac death (Emery, 2000). Muscle weakness usually appears during the second decade of life, sometimes after contractures occur (Bonne et al., 2000). Main difference between EDMD2 and LGMD1B can be found in wasting and weakness distribution (Maggi et al., 2014). As its name suggests, muscle weakness in LGMD1B is predominant in scapular and pelvic girdle areas in early stages (Muchir et al., 2000). Later, differential diagnosis between EDMD-2 and LGMD1B could be challenging with the progression of muscle weakness to the pelvic girdle in EDMD-2, which mimic LGMD1B phenotype. In these cases, identification of elbow contractures could support EDMD-2 diagnosis (Muchir et al., 2000).

*LMNA*-CMD phenotype had been described later: this diagnosis concerns children at birth or in their first two years of life (Quijano-Roy et al., 2008). Some of them display a severe congenital form with an absence of motor development. Most common phenotype is an axial muscle weakness with “dropped head syndrome” despite of a former normal acquisition of head carriage, a rigid spine and a scoliosis without locomotion loss (Quijano-Roy et al., 2008). Respiratory abnormalities are very frequent whereas cardiac features are rare, although sudden cardiac death could occur during the first ten years of life (Quijano-Roy et al., 2008). Analysis of an Italian cohort of *LMNA*-RD patients had confirmed cardiac involvement as it is the main feature in the natural history of patients phenotypes and cardiac follow-up is very important (Maggi et al., 2014). Usually, arrhythmia is the first cardiac symptom with electrocardiogram abnormalities such as low P-wave (atrial depolarization) or elongated PR-interval (period between onset of P-wave and ventricular depolarization) associated with rhythm abnormalities such as sinus bradycardia, sick sinus syndrome, bundle branch block or atrioventricular block or ectopic beats (Fatkin et al., 1999; Nigro et al., 2012). These symptoms appear mainly after the third decade of life (van Berlo et al., 2005).

## Mutations Pathogenesis Hypothesis

Nesprins and lamins both localize at NE and are ubiquitously expressed. However, mutations in genes encoding those proteins are responsible of tissue-specific diseases. Thus, several non-exclusive hypotheses have been proposed to explain the physiopathological mechanisms underlying these described diseases (**Figure 1C**).

### Structural Hypothesis

Structural hypothesis is based on “structural function” of the LINC complex and its ability to couple nucleoskeleton with cytoskeleton and associated interactors such as molecular motors and microtubule-associated proteins (MAPs), microtubules and actin network. LINC complex impairments could modulate extracellular transmission forces balance to the nucleus and cells could suffer from this induced mechanical damages (Zhang et al., 2007a; Banerjee et al., 2014; Bertrand et al., 2014). Nesprins have been shown to play a crucial role in myonuclei positioning and migration in muscles. It has been shown in *Caenorhabditis elegans* (Starr and Fridolfsson, 2010), in *Drosophila melanogaster* (Yu et al., 2006) but also in higher organisms (Wilson and Holzbaur, 2015; Gimpel et al., 2017; Zhou et al., 2017) that mutated nesprins (or orthologues) are responsible for mislocated nuclei mediated by an impaired recruitment of different partners such as Akap450 or KLC-1/2.

### Gene Regulation Hypothesis

Gene regulation hypothesis suggests that impaired LINC complex could alter interaction of NE proteins (mainly lamins) with the chromatin. Thus, mutations in genes encoding LINC complex proteins could alter in a tissue-specific manner transcription factors expression and/or alter expression pattern of tissue-specific genes (Dialynas et al., 2010). For example, such alterations are more and more described for laminopathies with the hyperactivation of the ERK (extracellular signal-regulated kinase) pathway in cardiomyopathy caused by *LMNA* mutations (Chatzifrangkeskou et al., 2018) or the overexpression of SMAD6, an inhibitor of the BMP signaling pathways, as a cause of accelerated myogenic differentiation of *LMNA* mutated cells (Janin et al., 2018).

Lamin A/C plays also a crucial role in euchromatin and heterochromatin distribution (Solovei et al., 2013) and in chromatin mobility restriction (Bronshtein et al., 2015). This chromatin interaction pathway exhibits a cell-type specific and differentiation-dependent pattern (Rønningen et al., 2015). Overexpression of wild-type lamin A/C, or mutated lamins related to progeroid syndromes (L647R) (Wang et al., 2016) or to FPLD and L-CMD (R388P) (Barateau et al., 2017) leads to different genes-lamins interactions patterns revealed using ChIP-seq analysis (Paulsen et al., 2017). These modifications are responsible for a more global three dimensional rearrangement of chromatin conformation (Oldenburg et al., 2014), associated with modification of epigenetic state of cells (Cesarini et al., 2015) and thus as a direct modification in gene expressions as it has been shown in FPLD-causing *LMNA* mutation R482W (Stierlé et al., 2003; Vadrot et al., 2015; Oldenburg et al., 2017).

Evidences support the epigenetic signature modifications hypothesis in cells. First, in patients suffering from HGPS, fibroblasts level of H3K27me3, a histone methylation often associated with the downregulation of nearby genes, is reduced and caused by a decrease level of the H3K27 methyltransferase of the PRC2 complex, called EZH2 (Shumaker et al., 2006). Interestingly, PRC2 complex regulates expression of *P16/INK4A* and *P21*, two tumor suppressors genes (Tzatsos et al., 2011), potentially filling the blank in understanding premature senescence observed in laminopathies (Guénantin et al., 2014). Finally, EZH2 plays a crucial role in the regulation of muscle gene expression and skeletal muscle differentiation (Caretti et al., 2004) and requires a functional lamin A/C network (Cesarini et al., 2015). These findings help to better understand how a ubiquitously expressed mutated protein could lead to tissue-specific phenotypes.

## Conclusion

The NE not only isolates genetic material from the rest of the cell, but also plays a crucial role in mechanotransduction signaling through the LINC complex that builds a bridge between cytoskeleton and nucleoskeleton. Moreover, interactions between chromatin, inner nuclear membrane and nuclear lamina are important to regulate gene expression. Thus, NE is recognized as a central complex organizing communication between genome and cytoskeleton, and most importantly as a key factor to mediate appropriate responses regarding external mechanical forces applied on cells.

In the past decade, LINC complex components and interactors have been identified either covering inner surface of the nucleus, or located directly inside inner or outer nuclear membrane. In parallel, mutations in genes encoding NE components have been associated with rare human diseases affecting numerous different tissues; from them, nesprin- and lamin-associated muscular diseases have emerged. Despite databases and large-scale studies recapitulating plenty of mutations, no clear correlation between a given genotype and its affected tissues or disease has been established. For example, *LMNA* mutations can lead to diseases affecting peripheral nerve, adipose tissue, skeletal muscle or heart, making the molecular diagnosis of envelopathies particularly challenging. At the same time, our progressive knowledge of nesprins and lamins roles at the NE, in the LINC complex organization, in regulation of gene expression, especially during myogenesis allows a better understanding of physiopathological mechanisms beneath described muscular and cardiac phenotypes. A complete understanding of different nesprins isoforms functions associated with an overview of proteins interacting network of lamins and nesprins is currently lacking.

Extensive use of Next Generation Sequencing (NGS) tools and of Whole Genome and Exome Sequencing (WGS and WES) in diagnosis laboratories will improve this discovery process. These tools, associated with the democratization of induced pluripotent stem cells (hiPSC) derived from affected patients or from healthy donor coupled with CRISPR-Cas9 technology in research laboratories, will also help to decipher the pathophysiological mechanisms underlying rare diseases without molecular explanation. Finally, taken together, these findings enable development of potential personalized therapeutic targets.

## Author Contributions

All authors wrote, read, and approved the manuscript.

## Conflict of Interest Statement

The authors declare that the research was conducted in the absence of any commercial or financial relationships that could be construed as a potential conflict of interest. The reviewer JR and handling editor declared their shared affiliation at the time of the review.

## Abbreviations

ABD: actin-binding domain
AD-EDMD: autosomal-dominant emery-dreifuss muscular dystrophy
AD: adaptive domain
AMC: arthrogryposis multiplex congenital
ARCA1: autosomal recessive cerebellar ataxia type 1
BMP: bone morphogenetic proteins
CH: calponin homology
CMD: congenital muscular dystrophy
CMT2B1: charcot-marie-tooth syndrome 2B1
CNS: central nervous system
DCM-CD: dilated cardiomyopathy phenotype with conduction defects
DCM: dilated cardiomyopathy
EDMD: emery-dreifuss muscular dystrophy
ERK: extracellular signal-regulated kinase
EZH2: enhancer of zeste homolog 2
FPLD: dunningan-type familial partial lipodystrophy
GWAS: genome-wide association studies
HGPS: hutchinson-gilford progeria syndrome
INM: inner nuclear membrane
KASH: Klarsicht/Anc-1/Syne Homology
KLC: kinesin light chain (KLC-1/2)
LGMD1B: limb-girdle muscular dystrophy 1B
LINC complex: linker of nucleoskeleton to cytoskeleton
LRMP: lymphoid restricted membrane protein
MAP: microtubule-associated proteins
MAPK: mitogen-activated protein kinases
MTOC: microtubule organizing center
NE: nuclear envelope
NGS: next generation sequencing
NPC: nuclear pore complex
ONM: outer nuclear membrane
PRC2: polycomb repressive complex 2
SR: spectrin repeats
SUN: Sad1/UNC-84 homology
TGF: tissue growth factor
VSMC: vascular smooth muscle cells
WES: whole exome sequencing
WGS: whole genome sequencing
YAP: yes-associated protein
